# Utilizing Endoscopy for the Diagnosis of Acute Upper Gastrointestinal Bleeding

**DOI:** 10.7759/cureus.40994

**Published:** 2023-06-26

**Authors:** Muhammad Z Akhtar, Moeen U Huq, Rahul Adwani, Ali Usman, Sarmad Ijaz, Iqra Seher

**Affiliations:** 1 Department of Medicine, Mayo Hospital, Lahore, PAK; 2 Department of Gastroenterology, Gomal Medical College, Dera Ismail Khan, PAK; 3 Department of Medicine, Dow University of Health Sciences, Civil Hospital Karachi, Karachi, PAK; 4 Department of Medicine, Shaikh Khalifa Bin Zayed Al-Nahyan Medical and Dental College, Lahore, PAK; 5 Department of Medicine, Avicenna Medical College, Lahore, PAK

**Keywords:** upper gastrointestinal (ugi) bleeding, upper gi endoscope, git endoscopy, esophageal and gastric varices, acute gastrointestinal bleed

## Abstract

Background

Acute upper gastrointestinal bleeding (UGIB) is a medical emergency requiring immediate diagnosis. While endoscopy is a commonly employed procedure in the evaluation of UGIB, its timing, outcomes, and the range of identified causes vary widely across different medical settings and regions. Therefore, the purpose of this study was to use endoscopy to investigate the cause of UGIB.

Methodology

A cross-sectional study was conducted at the Department of Gastroenterology, Mayo Hospital, Lahore, over a period of one year, from July 1st, 2021 to June 28th, 2022. The study enrolled all patients who were 18 years of age or older and exhibited symptoms of UGIB, including hematemesis and/or melena, within 48 hours of onset. An upper gastrointestinal endoscopy procedure was conducted in order to identify the underlying cause of UGIB and to apply appropriate therapeutic interventions. In patients where the endoscopic examination revealed bleeding ulcers, a specimen for biopsy was excised to test for Helicobacter pylori. Similarly, in cases where a malignancy was suspected during the endoscopy, a biopsy was performed for confirmatory diagnosis. A pre-designed proforma was utilized to collect data including the demographic variables such as age, gender, ethnicity, family history; clinical variables such as clinical presentation, comorbidities, medical history, medication use, vital signs, biochemical evaluation, and imaging results; endoscopic findings such as endoscopic location and severity of bleeding, endoscopic diagnosis, and the use of endoscopic interventions. Information relevant to the treatment and outcomes was also observed. Under outcomes, the rates of re-bleeding, need for repeat endoscopy, length of hospital stay, and mortality were recorded.

Results

The study reports that the mean age of the participants was 54.72 years with a standard deviation of 12.5 years. The mean hemoglobin level at the presentation was 7.98 ± 2.88 mg/dl. Out of the 309 patients, 215 (69.58%) were male, 202 (65.37%) presented with hematemesis, 97 (31.39%) presented with melena, and 10 patients had a mixed presentation. A total of 154 (49.84%) patients had portal hypertension. Out of these, 128 (83.12%) had esophageal varices and 21 (13.64%) had gastric varices. Five patients suffered from portal hypertensive gastropathy. In 114 (36.89%) patients, the cause of bleeding was ulcerative disease and out of these, duodenal ulcers were found in 49 (42.98%) while gastric ulcers were found in 22 (19.30%) patients. In total malignant lesions were detected in 20 (6.47%) cases.

Conclusion

The research indicates that hematemesis was the predominant initial symptom observed in individuals experiencing UGIB. The predominant etiology of the hemorrhage was identified as esophageal and gastric varices through endoscopic assessment. The study highlights the importance of early endoscopic evaluation in patients with UGIB as it can help identify the cause and guide appropriate management. This emphasizes the need for healthcare providers to be vigilant in identifying and managing patients with UGIB promptly to improve outcomes. Further research is needed to explore effective strategies for early detection and management of UGIB.

## Introduction

Acute upper gastrointestinal bleeding (UGIB) is a critical medical situation that arises when there is bleeding from a source proximal to the ligament of Treitz [1]. This condition is a leading cause of morbidity and mortality, with death rates ranging from 5-14% and increasing to over 40% in patients with liver disease [2]. Symptoms of acute UGIB may include anemia, hypovolemia, hematemesis, melena, or hematochezia [3].

Neoplasms, arteriovenous malformations, esophageal varices, gastric ulcers, gastric erosions, duodenal ulcers, reflux esophagitis, Mallory-Weiss tears, and fundal varices are just a few of the conditions that can cause acute UGIB [4]. Esophageal varices are the most frequent factor contributing to high prevalence of UGIB in our setting, while in Western countries, peptic ulcers are the leading cause [5-7]. Reports from different parts of the world indicate that acute UGIB has many causes that vary from country to country and even within geographical areas of the same country [4,5]. Peptic ulcer accounts for 50% of hospital admissions, while gastric erosions account for 10-20% of cases as reflected by a study from Rahim Yar Khan, Pakistan [4].

The prevailing factors that lead to UGIB in Pakistan, as per studies published within the local domain, include duodenal ulcers, gastric ulcers, esophageal varices, gastric erosion, esophagitis, Mallory-Weiss tears, arteriovenous malformation, and malignant lesions [4-8]. The incidence of UGIB is less common before the age of 20 and after the age of 60, with the highest occurrence among patients between the ages of 40-60 years, and males are more affected than females [5].

The research conducted by Wuerth and Rockey [6], had the objective of examining the evolving epidemiology of UGIB over the past ten years. Findings of the study indicate a noteworthy reduction in the frequency of UGIB, with a decrease from 92.1 to 70.2 per 100,000 hospitalizations during the period spanning from 2006 to 2014. The research additionally observed a reduction in the proportion of individuals necessitating blood transfusions, declining from 18.2% to 14.9% over the corresponding timeframe. Furthermore, the study also reported a shift in the etiology of UGIB, with a decrease in the proportion of patients with peptic ulcers and an increase in the proportion of patients with portal hypertension-related bleeding [6]. The study also revealed that individuals diagnosed with UGIB exhibited a higher probability of possessing coexisting medical conditions such as hepatic disorder, renal insufficiency, and cardiovascular ailment [6].

There could be various factors for the reduction in the occurrence of UGIB. Additionally, there may be an increased use of proton pump inhibitors (PPIs) and other medications for the prevention of peptic ulcers, which could reduce the incidence of UGIB. Most importantly, advances in diagnostic techniques and endoscopic therapies may have resulted in earlier detection and treatment of UGIB, which could have led to a reduction in the incidence and severity of the condition. Upper GI endoscopy is considered the primary modality for identifying the underlying etiology and administering appropriate therapeutic measures to arrest bleeding [9,10]. The authors Piazza et al. emphasized the significance of endoscopy in the treatment of UGIB. The authors have suggested that patients with high-risk features, such as active bleeding, stigmata of recent hemorrhage, or hemodynamic instability, should undergo early endoscopy within 24 hours of admission [10]. The authors also noted that endoscopic interventions such as thermal therapy, injection therapy, and mechanical hemostasis were effective in achieving hemostasis in up to 95% of cases [10].

In Pakistan, there is a relative scarcity of comprehensive data on UGIB, specifically concerning the diagnostic utility of endoscopy. This scarcity of information prompts an urgent need for context-specific data to improve the understanding and management of UGIB in the local healthcare context. Consequently, our study aimed to contribute to filling this knowledge gap by exploring the etiology of acute UGIB using upper gastrointestinal endoscopy in individuals presenting with acute gastrointestinal bleeding at a tertiary care center.

## Materials and methods

A cross-sectional study was undertaken at the Department of Gastroenterology, Mayo Hospital, Lahore between 1st July 2021 and 28th June 2022. All patients were recruited using a non-probability convenience sampling technique. Ethical approval was obtained from the institutional review board of Mayo Hospital, Lahore, with reference # IRB-MED-2922. During the study, all patients aged 18 years or older who presented with symptoms of upper gastrointestinal bleeding (UGIB) such as hematemesis and/or melena and within 48 hours of onset were included in the study. All cases in which patients were deemed unfit for endoscopy were excluded. Verbal and written informed consent was requested from all participants or their caretakers prior to their recruitment in the study.

The patients were either admitted to the department via the Emergency Room or the outpatient department. All patients with acute UGIB were immediately taken for resuscitation where the patient's vital signs were continuously monitored and were administered blood transfusions and intravenous fluids wherever necessary. After resuscitation, proper history and examination were conducted by the authors and baseline investigations were then advised.

Upper gastrointestinal endoscopy was performed to diagnose the cause of UGIB and application of therapeutic modality. In patients where the endoscopic examination revealed bleeding ulcers, a specimen for biopsy was excised to test for Helicobacter pylori. Similarly, in cases where a malignancy was suspected during the endoscopy, a biopsy was performed for confirmatory diagnosis. A predesigned proforma was utilized to collect data including demographic variables such as age, gender, ethnicity, family history; clinical variables such as clinical presentation, comorbidities, medical history, medication use, vital signs, biochemical evaluation, and imaging results; endoscopic findings such as endoscopic location and severity of bleeding, endoscopic diagnosis, and the use of endoscopic interventions. Information relevant to the treatment and outcomes was also observed. Under outcomes, the rates of re-bleeding, need for repeat endoscopy, length of hospital stay, and mortality were recorded.

The data analysis was conducted using the Statistical Package for Social Science (SPSS) version 24 (IBM Corp., Armonk, NY, USA). Categorical variables such as gender, etiology of upper gastrointestinal bleeding, rate of re-bleeding, and mortality were subjected to frequency and percentage computations. Descriptive statistics were calculated for the continuous variables, including age, hemoglobin, and platelets, in the form of mean and standard deviation.

## Results

**Figure 1:**
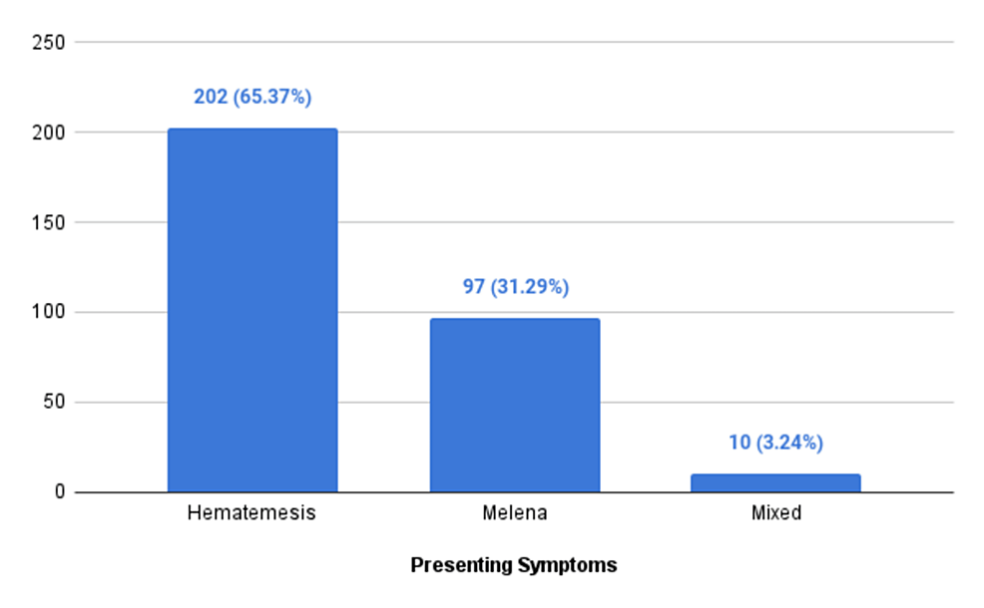
Distribution of study participants according to presenting symptom (N = 309)

The study comprised a sample of 309 patients who exhibited symptoms of upper gastrointestinal bleeding as illustrated in Figure 1. Out of the 309 patients, 202 (65.37%) presented with hematemesis, 97 (31.39%) presented with melena, and 10 patients had a mixed presentation (both melana and hematemesis).

**Figure 2:**
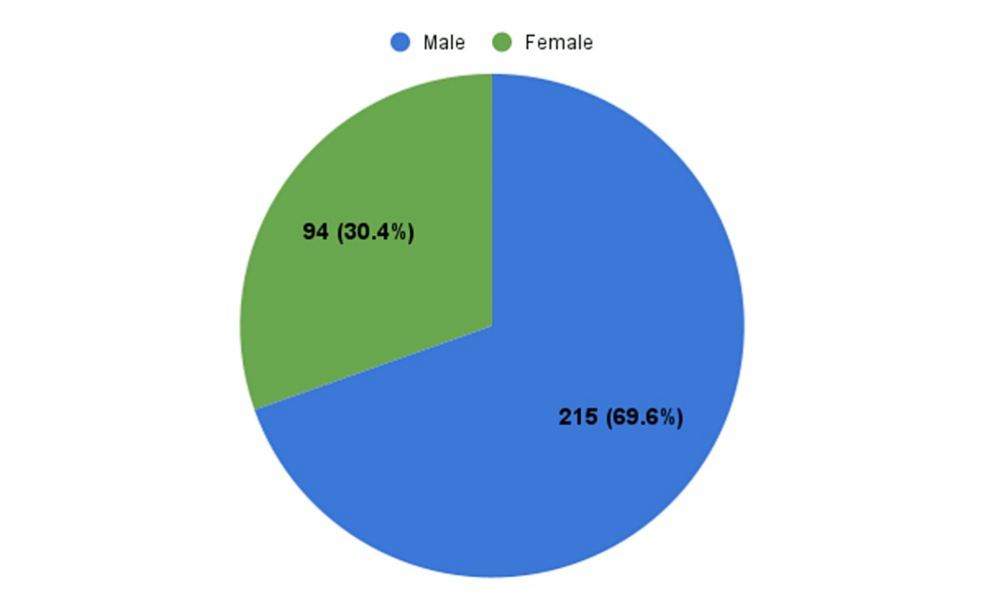
Distribution of study participants according to gender (N = 309)

The mean age of study participants was 54.72 years with a standard deviation of 12.5 years. The mean hemoglobin level at the presentation was 7.98 ± 2.88 mg/dl (male: 13.8 to 17.2 grams per deciliter (g/dl) and female: 12.1 to 15.1 g/dl). Out of the 309 patients, 215 (69.58%) were males as illustrated in Figure 2.

Among the 309 study participants, 154 (49.84%) had portal hypertension. Out of these 154 patients, 128 (83.12%) had esophageal varices, 21 (13.64%) had gastric varices, and five patients suffered from portal hypertensive gastropathy. Out of the total 309 patients, 114 (36.89%) patients had ulcerative disease. Out of these 114 patients, duodenal ulcers were found in 49 (42.98%) while gastric ulcers were found in 22 (19.30%) patients. In total malignant lesions were detected in 20 (6.47%) cases (Table 1).

**Table 1:** Causes of bleeding among the study population (N = 309) *a single study participant could have multiple causes of bleeding.

Cause of bleeding*	N (%)
Portal hypertension	154 (49.84%)
Esophageal varices	128 (83.12%)
Gastric varices	21 (13.64%)
Portal hypertensive gastropathy	5 (3.25%)
Ulcerative disease	114 (36.89%)
Duodenal ulcers	49 (42.98%)
Gastric ulcers	22 (19.30%)
Mixed lesions (both duodenal and gastric ulcers)	19 (16.67%)
Others (including ulcers on the esophageal, gastroesophageal junction, and jejunum)	24 (21.05%)
Malignant lesions	20 (6.47%)
Esophageal tumor	4 (20.00%)
Gastric tumor	14 (70.00%)
Duodenal tumor	2 (10.00%)
Miscellaneous lesions	28 (6.8%)

## Discussion

The results of our study are consistent with the current body of academic research. Jung and Moon have demonstrated the indispensability of endoscopy in the treatment of upper gastrointestinal bleeding (UGIB) owing to its ability to detect the bleeding source and facilitate the implementation of hemostatic interventions. According to the authors' findings, endoscopy demonstrated a success rate of over 90% in identifying the bleeding source, while endoscopic interventions were able to achieve hemostasis in up to 80% of cases. The timing of endoscopy was determined as a crucial aspect in the management of acute gastrointestinal bleeding, as highlighted by the authors. The performance of endoscopy at an early stage, specifically within 24 hours of admission, has been linked to favorable outcomes such as decreased mortality rates and reduced hospitalization durations. The authors recommended that early endoscopy should be performed in all cases of acute UGIB, unless contraindicated [11].

Another study by Shangavi et al. [12] revealed that the most common cause of UGIB was peptic ulcer disease, which accounted for 42.4% of cases. Other causes of UGIB included esophageal varices (22.4%), Mallory-Weiss tears (12.8%), and erosive gastritis (7.2%). The study also found that endoscopy was successful in identifying the source of bleeding in 89.6% of cases [12]. The authors noted that early endoscopy, within 24 hours of admission, was associated with improved outcomes, including reduced length of hospital stay and lower rates of re-bleeding [12].

In our study, all patients were offered endoscopy within 24 hours of admission. The current evidence regarding the benefit of early endoscopy is unclear. Some studies suggest mortality benefits, while others suggest otherwise [13]. While data suggest that there is a potential benefit in performing endoscopy sooner, there is no concrete evidence to point to a particular time frame [14].

According to previous research, males are more likely to experience upper gastrointestinal bleeding due to increased exposure to risk factors such as barber shaves, intravenous medication, and tattooing, as seen in studies conducted by Kar et al. [15], and Masood-Ur-Rahman et al. [16], in Peshawar, which reported male predominance. The leading cause of upper gastrointestinal bleeding in the current study was varices, which is in line with earlier research carried out in Pakistan by Khan et al. [17]. In contrast, Salma et al. assessed a total of 100 patients with UGIB, out of which 62% were males and 38% were females. The most common presenting symptom was hematemesis (71%), followed by melena (56%). Endoscopic evaluation revealed that peptic ulcer disease (40%) was the most common cause of UGIB, followed by esophageal varices (23%) and erosive gastritis (18%). Other causes included malignancy, Mallory-Weiss syndrome, and Dieulafoy’s lesion [18]. The study found that endoscopic evaluation was effective in identifying the cause of UGIB in most cases and provided valuable information for the management of patients. The authors recommended early endoscopic evaluation for patients with UGIB to improve diagnosis and management.

Despite the strengths of the present study, there were some limitations of the study. Firstly, a cross-sectional study cannot establish causality or make comparisons with other populations. Secondly, due to the convenience sampling technique, bias may have been introduced hence, limiting the generalizability of the findings to other populations. Thus, we recommend that further research using a multi-center approach be conducted to explore the topic in more detail.

## Conclusions

Our research determined that hematemesis was the predominant initial manifestation observed in individuals experiencing upper gastrointestinal bleeding (UGIB). The predominant etiology of the hemorrhage was identified as esophageal and gastric varices through endoscopic assessment. The study highlights the importance of early endoscopic evaluation in patients with UGIB as it can help identify the cause and guide appropriate management. This emphasizes the need for healthcare providers to be vigilant in identifying and managing patients with UGIB promptly to improve outcomes. Further research is needed to explore effective strategies for early detection and management of UGIB.
